# Genome-Wide Analyses of Calcium Sensors Reveal Their Involvement in Drought Stress Response and Storage Roots Deterioration after Harvest in Cassava

**DOI:** 10.3390/genes9040221

**Published:** 2018-04-19

**Authors:** Wei Hu, Yan Yan, Weiwei Tie, Zehong Ding, Chunlai Wu, Xupo Ding, Wenquan Wang, Zhiqiang Xia, Jianchun Guo, Ming Peng

**Affiliations:** Key Laboratory of Biology and Genetic Resources of Tropical Crops, Institute of Tropical Bioscience and Biotechnology, Chinese Academy of Tropical Agricultural Sciences, Xueyuan Road 4, Haikou 571101, Hainan, China; yanyan@itbb.org.cn (Y.Y.); tieweiwei@itbb.org.cn (W.T.); dingzehong@itbb.org.cn (Z.D.); wuchunlai19900109@126.com (C.W.); dingxupo@itbb.org.cn (X.D.); wangwenquan@itbb.org.cn (W.W.); xiazhiqiang@itbb.org.cn (Z.X.)

**Keywords:** calcium, calcium sensors, cassava, drought, genome-wide analysis, postharvest physiological deterioration

## Abstract

Calcium (Ca^2+^) plays a crucial role in plant development and responses to environmental stimuli. Currently, calmodulins (CaMs), calmodulin-like proteins (CMLs), and calcineurin B-like proteins (CBLs), such as Ca^2+^ sensors, are not well understood in cassava (*Manihot* *esculenta* Crantz), an important tropical crop. In the present study, 8 CaMs, 48 CMLs, and 9 CBLs were genome-wide identified in cassava, which were divided into two, four, and four groups, respectively, based on evolutionary relationship, protein motif, and gene structure analyses. Transcriptomic analysis revealed the expression diversity of cassava CaMs-CMLs-CBLs in distinct tissues and in response to drought stress in different genotypes. Generally, cassava CaMs-CMLs-CBLs showed different expression profiles between cultivated varieties (Arg7 and SC124) and wild ancestor (W14) after drought treatment. In addition, numerous CaMs-CMLs-CBLs were significantly upregulated at 6 h, 12 h, and 48 h after harvest, suggesting their possible role during storage roots (SR) deterioration. Further interaction network and co-expression analyses suggested that a CBL-mediated interaction network was widely involved in SR deterioration. Taken together, this study provides new insights into CaMs-CMLs-CBLs-mediated drought adaption and SR deterioration at the transcription level in cassava, and identifies some candidates for the genetic improvement of cassava.

## 1. Introduction

As a second messenger, calcium (Ca^2+^) plays a crucial role in various biological processes [1]. Cellular calcium concentrations are modulated in plants responding to signals, such as light, hormones, pathogens, and abiotic stress [2,3]. The transient cellular Ca^2+^ increase can be recognized and decoded by four classes of Ca^2+^ sensors, including calmodulins (CaMs), calmodulin-like proteins (CMLs), calcineurin B-like proteins (CBLs), and calcium-dependent protein kinases (CDPKs/CPKs), which results in the activation of downstream events, including protein phosphorylation and gene expression in various organisms [4]. All four types of Ca^2+^ sensors have the typical EF-hand domain that is responsible for Ca^2+^ binding.

Ca^2+^ sensors are widely involved in multiple processes related to plant growth and development, such as root hair elongation, guard cell regulation, pollen tube growth, and hormone signaling. Vacuolar CBL2/3-CIPK12 (CBL-interacting protein kinases) complexes are required for polarized pollen tube growth [5]. CBL2/3 also functions positively, affecting leaf, root, silique, and seed development and ion homeostasis through regulation of vacuolar-type H^+^-ATPase (V-ATPase) activity [6,7]. CML39 plays a positive role in transducing light signals and promoting early seedling establishment [8]. CBL1 and CBL9 regulate pollen germination and pollen tube growth through affecting K^+^ (potassium) homeostasis [9]. CBL1 also plays a role in plants responding to glucose and gibberellin signals during germination and seedling development [10]. Moreover, there is considerable evidence showing that Ca^2+^ sensors participate in the abiotic stress response by regulating abscisic acid (ABA) signaling, stomatal behavior, ion homeostasis, and osmotic balance. In *Arabidopsis*, many genes encoding Ca^2+^ sensors were confirmed to positively regulate plant tolerance of abiotic stress (osmotic, drought, cold, or salt), including CPK3, CPK4, CPK6, CPK10, CPK11, CPK21, CPK27, CPK32, CaM1, CaM3, CaM4, and CBL1 [11,12,13,14,15,16]. This is also validated in other plants [17,18]. In contrast, some Ca^2+^ sensors were found to negatively relate to abiotic stress response. *cml9* knock-out mutants showed enhanced tolerance of drought and salt stresses in *Arabidopsis* [19]. Overexpression and mutation of CPK23 revealed its negative role in tolerance to drought and salt stresses [20]. Loss-of-function of CPK21 resulted in increased tolerance to hyperosmotic stress [13]. These evidences indicate that the CDPK-mediated abiotic stress response is complex.

Currently, based on genome-wide analysis, Ca^2+^ sensors have been identified in various species [21,22,23,24,25,26]. There are 10 CBLs in *Arabidopsis* and 10 in rice, seven CaMs in *Arabidopsis* and five in rice, and 50 CMLs in *Arabidopsis* and 32 in rice. Previously, we identified 27 CPKs from cassava (*Manihot esculenta* Crantz) [24]. However, less information is known about other Ca^2+^ sensors (CBLs, CaMs, and CMLs) in cassava. As a staple food crop, cassava is the sixth most important crop after wheat, rice, maize, potato, and barley [27]. Its edible storage roots (SR) supply a source of dietary carbohydrate for over 600 million people worldwide [28]. Cassava is also considered as a potential biofuel crop for production of ethanol and bioenergy, due to its high starch production [27]. Moreover, cassava has strong tolerance of drought and low-fertility environments, due to its effective use of light, heat, and water resources. Notably, the potential of cassava as a food and industrial crop is largely restricted by the rapid post-harvest physiological deterioration of the SR, which starts within 72 h after harvest [29]. The mechanism underlying cassava tolerance of drought stress and sensitivity to post-harvest physiological deterioration are largely unknown.

Considering the importance of Ca^2+^ sensors in plant growth, development, and response to abiotic stresses, an effort was made to identify CBLs, CaMs, and CMLs from cassava, and investigate their phylogenetic relationships, protein motifs, gene structure, and expression profiles in distinct tissues, in response to drought stress and during SR deterioration. This systematic study would increase our understanding of Ca^2+^ sensors associated with drought responses and SR deterioration, and lays a foundation for the genetic improvement of cassava.

## 2. Materials and Methods

### 2.1. Plant Materials and Treatments

The characteristics of SC124, Arg7, and W14 cassava genotypes were described in previous studies [30,31]. W14 is an accession of the wild ancestor, and had stronger drought resistance than the cultivated varieties SC124 and Arg7 [31]. Segments of cassava stems from mother plants were cultured in pots filled with soil and vermiculite (1:1) in a growth room with a 16 h/35 °C day and 8 h/20 °C night regime, and a relative humidity of 70%. Thereafter, stems (90-day-old), leaves (90-day-old), and SR (150-day-old) were cut from Arg7 and W14 under growth room conditions, to study the expression levels of CaM-CML-CBLs in distinct organs. To detect the transcriptional changes of CaM-CML-CBLs in response to drought, 90-day-old cassava plants of Arg7, SC124, and W14 were subjected to water withholding for 12 days, and the leaves and roots were collected for RNA sequencing. To study the transcriptional changes of CaM-CML-CBLs during SR deterioration, 10-month-old cassava SR with 5–6 cm diameter stems were cut into slices approximately 5 mm thick, and then moved into petri dishes containing a wet filter paper [29]. After incubation at 28 °C and 60% relative humidity in the dark for 0 h, 6 h, 12 h, and 48 h, the slices were sampled and frozen in liquid nitrogen until the extraction of total RNA.

### 2.2. Identification and Phylogenetic Analyses

The whole protein sequences of cassava were downloaded from the cassava genome database [32]. The CaM, CML, and CBL protein sequences from rice and *Arabidopsis* were obtained from the rice genome annotation project (RGAP) and UniPort databases, respectively [33,34]. The hidden markov models (HMM) profiles built from CaMs, CMLs, and CBLs in *Arabidopsis* and rice were used as queries to identify the putative cassava CaMs, CMLs, and CBLs with HMMER software [35]. In addition, protein sequences of CaMs, CMLs, and CBLs from *Arabidopsis* and rice were used in a basic local alignment search tool (BLAST [36]) search against cassava proteins, to detect other CaMs, CMLs, and CBLs that might be missed by the HMM profile. After removing the redundant sequences, the conserved domain (EF-hand) of predicted cassava CaMs, CMLs, and CBLs were further confirmed with Pfam and conserved domains database (CDD) databases [37,38]. The accession number of identified cassava CaMs, CMLs, and CBLs is displayed in Table S1. The phylogenetic tree was created with the CaMs, CMLs, and CBLs from *Arabidopsis*, rice, and cassava using MEGA 5.0 and Clustal X2.0 softwares (bootstrap values for 1000 replicates) [39,40].

### 2.3. Protein Properties and Sequence Analyses

The isoelectric points and molecular weights of cassava CaMs, CMLs, and CBLs were predicted using ExPASy database [41]. The conserved motifs of cassava CaMs, CMLs, and CBLs were identified by MEME database, and were further annotated with InterProScan database [42,43]. The optimum width of motifs ranged from 6 to 50 and the maximum number of motifs was 5. The gene structures of cassava CaMs, CMLs, and CBLs were analyzed by gene structure display server (GSDS) [44]. The interaction network of CaM, CML, and CBL members were built by STRING database [45], with interaction score > 0.7 and no more than 20 interactors.

### 2.4. Transcriptomic Analysis

The total RNA of each sample, extracted with a plant RNA extraction kit (DP432, TIANGEN Biotech, Beijing, China), was used to construct complementary DNA (cDNA) libraries. The sequencing was performed with an Illumina GAII (Illumina, San Diego, CA, USA), following the manufacturer’s instructions. Single-end libraries (non-strand-specific) were sequenced with a read length of 51 bp. Adapter sequences in the raw sequence reads were removed using the FASTX-toolkit [46]. After examining the sequence quality and removing low quality sequences by FastQC [47], clean reads were generated. Using TopHat v2.0.10, clean reads were mapped to the cassava reference genome (v4.1) [48]. The transcriptome assemblies were performed by Cufflinks [49]. Gene expression levels were calculated as fragments per kilobase of transcript per million fragments mapped (FPKM). Differentially expressed genes between treatments (after drought or after harvest) and control were identified based on absolute value of Log_2_ based fold changes > 1. The accession number of transcriptomic data was listed in Table S1.

## 3. Results

### 3.1. Genome-Wide Identification and Evolutionary Analyses of the Calmodulins (CaMs), Calmodulin-Like Proteins (CMLs), and Calcineurin B-Like Proteins (CBLs) in Cassava

Previously, we identified 27 calcium-dependent protein kinases and one type of calcium sensor from the cassava genome [24]. Here, a total of eight CaM, 48 CML, and nine CBL proteins were identified from the cassava genome, based on HMM and BLAST searches. The predicted CaM, CML, and CBL proteins varied from 84 to 318 amino acid residues, with relative molecular masses in the range of 9.3–34.9 kDa, and theoretical isoelectric points (PIs) ranging from 3.9 to 7.0 (Table S2).

**Figure 1:**
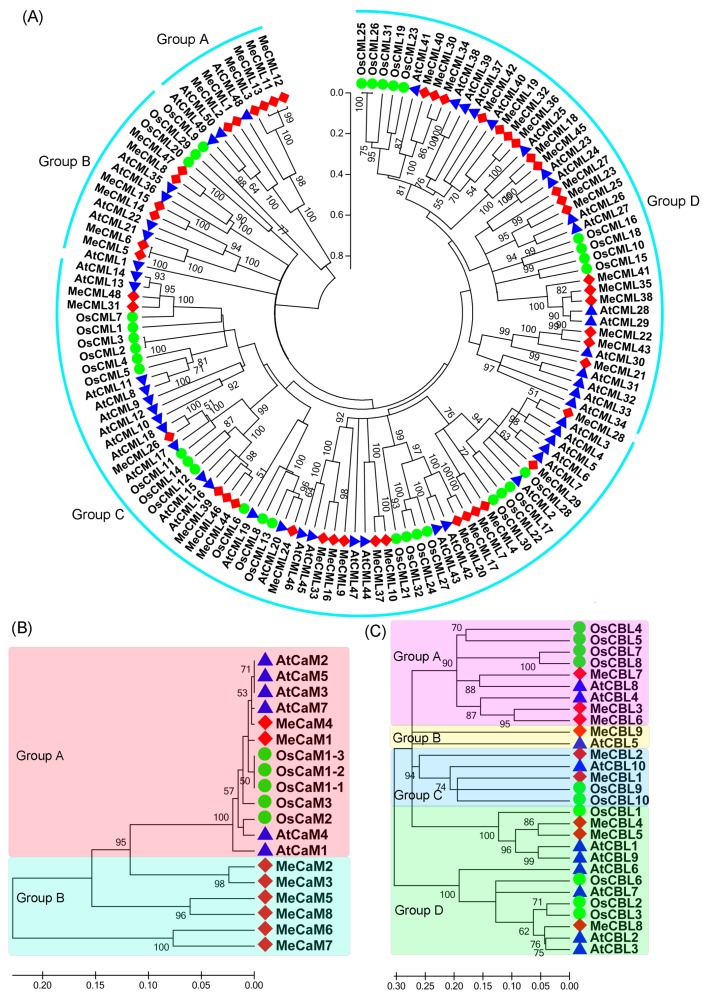
Phylogenetic analysis of calmodulin-like proteins (CMLs) (**A**), calmodulins (CaMs) (**B**), and calcineurin B-like proteins (CBLs) (**C**) from cassava, *Arabidopsis*, and rice. The Neighbor-joining (NJ) tree was constructed using Clustal X 2.0 and MEGA 5.0 software with the pair-wise deletion option. One thousand bootstrap replicates were used to assess tree reliability. Blue triangle, calcium (Ca^2+^) sensors in *Arabidopsis*; Red square, Ca^2+^ sensors in cassava; Green circle, Ca^2+^ sensors in rice. Shade areas with different colors indicate distinct groups of CaM and CBL families.

To understand the phylogenetic relationship of CaM, CML, and CBL proteins, neighbor-joining (NJ) trees were created with CaM, CML, and CBL proteins from cassava, *Arabidopsis* and rice (Figure 1; Table S3). The cassava CaM, CML, and CBL families were classified into 2 (group A-B), 4 (group A-D), and 4 (group A-D) groups, respectively, based on their phylogenetic relationships. Generally, cassava CaMs, CMLs, and CBLs showed closer relationships with those in *Arabidopsis* than those in rice, supported by the orthologous genes between *Arabidopsis* and cassava.

### 3.2. Conserved Motifs and Gene Structure Analyses of calmodulins (CaMs), Calmodulin-Like Proteins (CMLs), and Calcineurin B-Like Proteins (CBLs) in Cassava

**Figure 2:**
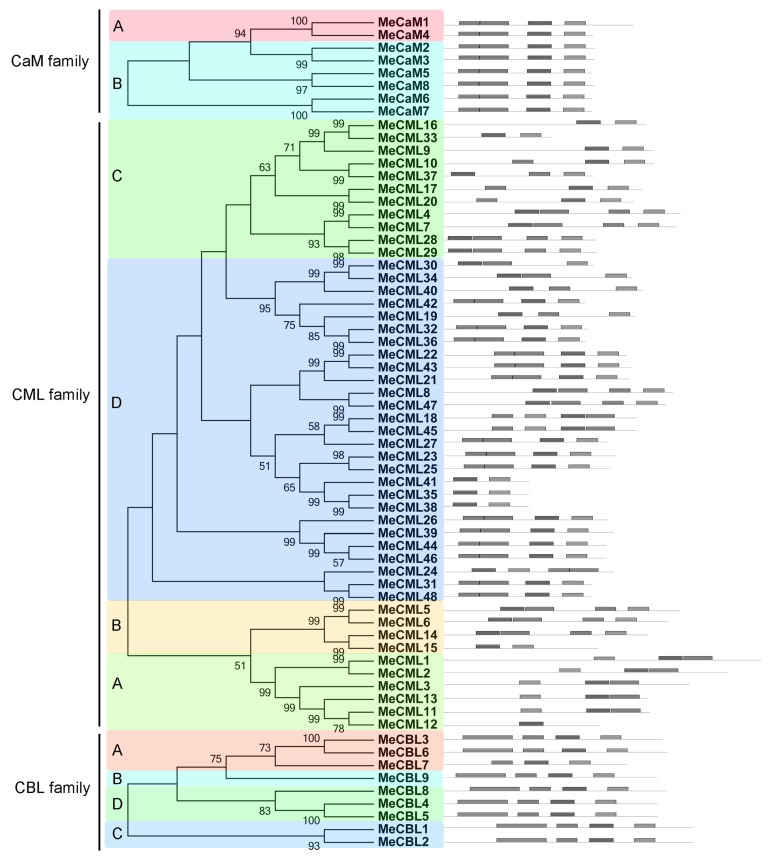
The conserved motifs of cassava CaMs, CMLs, and CBLs according to phylogenetic relationship. All the grey boxes indicate EF-hand domains for each protein. All motifs were identified by MEME database with the complete amino acid sequences of cassava CaMs, CMLs, and CBLs. Each family showed similar motif organization. Shade areas with different colors indicate distinct groups of CaM, CML, and CBL families.

To investigate structural features of the cassava CaMs, CMLs, and CBLs, conserved motifs were analyzed based on the phylogenetic relationship. Four conserved motifs that were annotated as EF-hand domains for the CaM, CML, and CBL families were acquired with MEME and InterPro databases (Figure 2). For the CaM family, all the members contain four4 EF-hand domains. For the CML family, groups A, B, C, and D have 1–3, 2–4, 2–4 and 2–4 EF-hand domains, respectively. For the CBL family, all the members show four EF-hand domains, except for MeCBL7, which has three EF-hand domains. Based on the above results, all the identified CaMs, CMLs, and CBLs had at least one EF-hand domain, indicating their typical family features of calcium binding.

**Figure 3:**
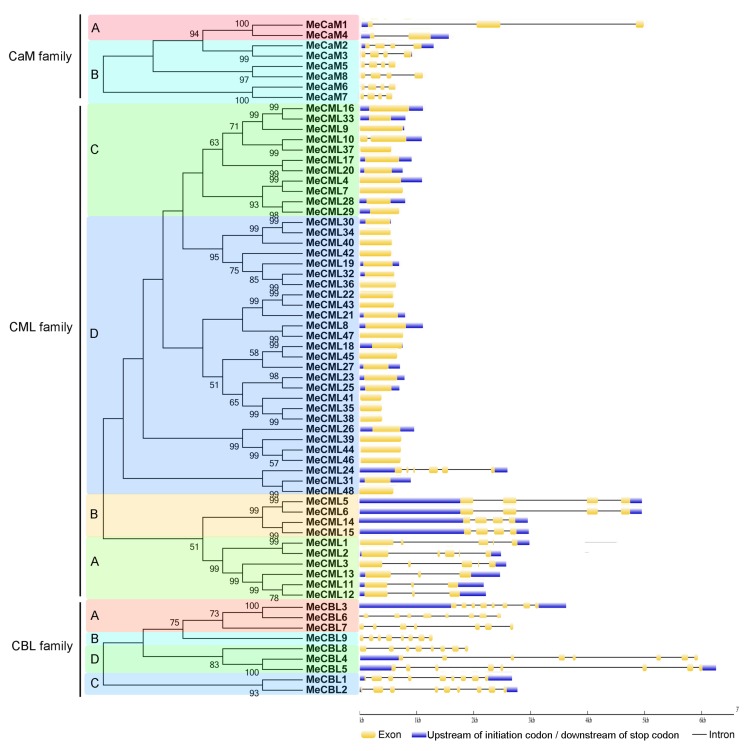
Gene structure analyses of cassava CaMs, CMLs, and CBLs according to phylogenetic relationship. Exon-intron structure analyses were performed through the gene structure display server database. The blue boxes, yellow boxes, and black lines indicate upstream of initiation codon/downstream of stop codon, exons, and introns, respectively. Shade areas with different colors indicate distinct groups of CaM, CML, and CBL families.

Additionally, the exon-intron organizations of these genes were tested using the GSDS database (Figure 3). For the CaM family, group A shows two to three exons and group B contains four exons. For the CML family, group A shows three to five exons, group B has four exons, group C has one to two exons, and group D displays one exon, except for *MeCML24*, with six exons. For the CBL family, groups A, B, C, and D contain seven to eight, eight, nine, and eight exons, respectively. Generally, the proteins classified into the same subgroup share similar motifs and exon-intron organizations, suggesting a link between evolution and conserved motifs/gene structure.

### 3.3. Expression Analyses of Calmodulins (CaMs), Calmodulin-Like Proteins (CMLs), and Calcineurin B-Like Proteins (CBLs) in Different Cassava Tissues

**Figure 4:**
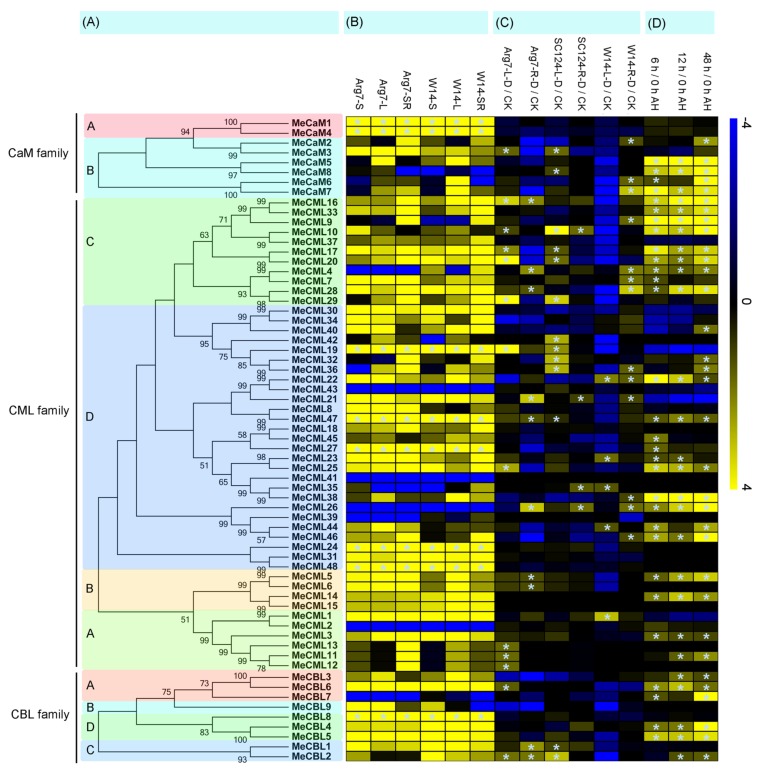
Expression profiles of CaMs, CMLs, and CBLs in cassava, according to phylogenetic relationship. (**A**) Phylogenetic relationship of cassava CaMs, CMLs, and CBLs. (**B**) Expression profiles of cassava CaMs, CMLs, and CBLs in stems (90-day-old), leaves (90-day-old), and storage roots (150-day-old) of Arg7 and W14. Asterisks indicate the genes that show high expression levels (Log_2_ based fragments per kilobase of transcript per million fragments mapped (FPKM) > 5) in all the tissues examined. S, stem; L, leaf; SR, storage root. (**C**) Expression profiles of cassava CaMs, CMLs, and CBLs in response to drought stress in leaves and roots of Arg7, SC124, and W14. Ninety-day-old cassava plants were subjected to water withholding for 12 days, and leaves and roots were collected for RNA-seq. Asterisks indicate the genes that are induced (Log_2_ based fold changes > 1) after drought treatment. L, leaf; R, root; D, drought treatment; CK, control check showing leaves or roots of cassava varieties under normal conditions. (**D**) Expression profiles of cassava CaMs, CMLs, and CBLs in cross-sessions of SR at 6 h, 12 h, and 48 h compared with 0 h after harvest. Ten-month-old storage roots were cut into slices approximately 5 mm thick, and then were moved into petri dishes containing a wet filter paper. After incubation at 28 °C and 60% relative humidity in the dark for 0 h, 6 h, 12 h, and 48 h, the slices were sampled for RNA-seq. Asterisks indicate the genes that is induced (Log_2_ based fold changes > 1) after harvest. AH, after harvest. Log_2_ based FPKM (**B**) or fold change (**C**) and (**D**) was used to create the heat map. Changes in gene expression are shown in color as the scale.

To study the expression profiles of CaMs, CMLs, and CBLs in different tissues, transcriptome analyses were carried out from samples of leaves, stems, and storage roots in wild ancestor (W14) and cultivated variety (Arg7) (Figure 4; Table S4). Generally, most of the CaMs, CMLs, and CBLs had similar expression profiles in different tissues between Arg7 and W14. For example, several genes (*MeCaM-1*, *-4*, *MeCML-19 -24*, *-27*, *-47*, *-48 and MeCBL-8*) showed high expression levels (Log_2_ FPKM value > 5) in various tissues of Arg7 and W14. In contrast, some genes (*MeCaM-8*, *MeCML-1*, *-26*, *-41*, *-43* and *MeCBL-7*) had low transcript abundance (Log_2_ FPKM value < 1) in both Arg7 and W14.

Additionally, some CaM, CML, and CBL genes showed different expression profiles in different genotypes. *MeCaM3* and *MeCML-8*, *-22*, *-23*, *-40* showed high expression levels (Log_2_ FPKM > 5) in Arg7 stem, but low expression (Log_2_ FPKM < 3) in W14 stem. *MeCaM-6*, *-7* had low transcript abundance (Log_2_ FPKM < 3) in Arg7 leaf, but high expression (Log_2_ FPKM > 5) in W14 leaf. *MeCML-8*, *-11*, *-12*, *-13*, *-36* had high expression (Log_2_ FPKM > 5) in Arg7 storage root, but low expression (Log FPKM < 3) in W14 storage root. Together, the tissue expression profiles of CaMs, CMLs, and CBLs would be benefit for further study of tissue development and function.

### 3.4. Expression Analyses of Calmodulins (CaMs), Calmodulin-Like Proteins (CMLs), and Calcineurin B-Like Proteins (CBLs) Genes in Response to Drought Stress

Since previous reports revealed that the CaM, CML, and CBL families could participate in osmotic adjustment and drought stress response, the expression profiles of CaMs, CMLs, and CBLs in response to drought stress were further detected in three cassava genotypes by transcriptome analysis (Figure 4; Table S5). Totally, 14 and 10 *MeCaMs-MeCMLs-MeCBLs* showed induction in leaf and root of Arg7, respectively. The corresponding number is 12 and four in SC124, and five and 13 in W14. From the above results, we found that: (i) the total number of *MeCaMs-MeCMLs-MeCBLs* induced by drought is greater in Arg7 than in SC124 and W14; (ii) the number of *MeCaMs-MeCMLs-MeCBLs* induced by drought was greater in roots than in leaves in W14, whereas it was reduced in roots compared with leaves in Arg7 and SC124.

### 3.5. Expression Patterns of Cassava Calmodulins (CaMs), Calmodulin-Like Proteins (CMLs), and Calcineurin B-Like Proteins (CBLs) after Harvest

Transcriptomic analysis was performed to study the expression profiles of cassava CaMs, CMLs, and CBLs after harvest (Figure 4; Table S6). In comparison to cross-sessions of SR at 0 h after harvest, 38, 38, and 42 calcium sensor genes showed up-regulation at 6 h, 12 h, and 48 h after harvest, respectively, among which 30, 27, and 33 genes showed significant induction (Log_2_ based fold change > 1) at 6 h, 12 h, and 48 h after harvest, respectively. Moreover, 23 genes had significantly increased transcripts (Log_2_ based fold change > 1) during all the tested stages after harvest. Together, these results suggested the possible involvement of calcium signaling in the regulation of cassava SR deterioration.

### 3.6. Calmodulins (CaMs), Calmodulin-Like Proteins (CMLs), and Calcineurin B-Like Proteins (CBLs) Interaction Networks and Their Co-Expression after Harvest

**Figure 5:**
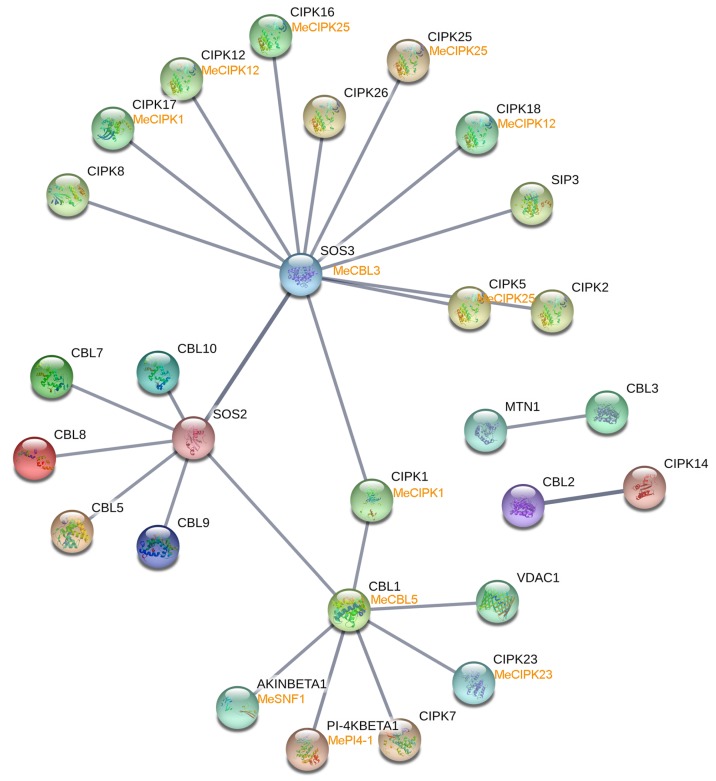
Interaction network and co-expression analyses of CBLs at 48 h/0 h in cross-sessions of SR after harvest. The interacting relationship between CBLs and other proteins are indicated by the gray lines. The genes at upper and lower around each ball show CBL-mediated interaction network in *Arabidopsis* and cassava, respectively. The gene pairs that showed uniform upregulation (Log_2_ based fold change > 1) at 48 h/0 h after harvest in cassava were marked with yellow.

To better understand the function of cassava CaM-CML-CBLs, the interaction networks and co-expression profiles of these proteins were detected according to experimentally validated interactions of CaM-CML-CBLs in *Arabidopsis* (Figure 5; Tables S7–S12). Firstly, CaM-, CML-, and CBL-mediated interaction networks in *Arabidopsis* were built using STRING database (Tables S7–S9). Then, the homologs of these proteins in the interaction network were identified in cassava using the reciprocal BLAST protein analysis (Table S10). Finally, the expression profiles of these cassava genes at 0 h and 48 h after harvest were searched from our previous RNA-seq. data sets. The gene pairs showing upregulation (Log_2_ based fold change > 1) or downregulation (Log_2_ based fold change < −1) at 48 h/0 h after harvest were displayed in Table S10.

For the CaM-mediated interaction network, no gene pair displayed co-expression (Figure S1A; Table S11). For the CML-mediated interaction network, only one gene pair (*AT2G27480:Me014830-AT1G15130:Me001472*) showed uniform upregulation (Figure S1B; Table S11). For the CBL-mediated interaction network, *SOS3/MeCBL3*-mediated seven gene pairs and *CBL1/MeCBL5*-mediated four gene pairs had uniform upregulation (Figure 5; Tables S11 and S12). These genes included eight CBL-interacting protein kinases (CIPKs), one phosphatidylinositol 4-kinase (PI-4KBETA1), and one SNF1-related protein kinase (AKINBETA1) (Table S10). These results indicated that CBL-mediated interaction and co-expression network may play a crucial role during cassava SR deterioration.

## 4. Discussion

Calcium, as an important second messenger, plays crucial roles in various aspects of biological processes in plants. Calcium signals are sensed and decoded by Ca^2+^ sensors, including CaMs, CMLs, CBLs, and CPKs [24,30]. Previously, we identified 27 CPKs from the cassava genome [24]. Here, eight *MeCaMs*, 48 *MeCMLs*, and nine *MeCBLs* were identified from the cassava genome, and their phylogenetic relationships, gene structures, and conserved motifs were investigated (Figure 1, Figure 2 and Figure 3). Phylogenetic analyses showed that cassava CaMs, CMLs, and CBLs could be divided into two, four and four groups, respectively. together with those in *Arabidopsis* and rice (Figure 1), which is consistent with previous phylogenetic classification of CaMs, CMLs, and CBLs in *Arabidopsis*, rice, strawberry, cotton, grapevine, and *Lotus japonicus* [22,23,25,50]. Moreover, this classification was also validated by conserved motif analysis, which indicated that all the identified CaMs, CMLs, and CBLs contained the typical EF-hand, and each subfamily shared similar motifs and exon-intron organizations (Figure 2 and Figure 3). The typical EF-hand organizations of CaMs, CMLs, and CBLs in cassava were the same as those in other plant species. The CaMs strictly contain four typical EF-hand domains in various plant species [22,25,51]; the CMLs generally had variable numbers of EF-hands from one to six [22,25,51]; and the CBLs generally had four EF-hands [21,23]. Thus, the conserved EF-hand organizations of CaMs, CMLs, and CBLs are conserved across plant species. Interestingly, CaMs in group B from cassava apparently separated from *Arabidopsis* and rice with strong bootstrap support, indicating an independent evolution of these CaMs in cassava relative to *Arabidopsis* and rice (Figure 1). This group of cassava CaMs showed similar conserved motifs and exon-intron organization, implying their consistent origin (Figure 2 and Figure 3). Notably, cassava CaMs in group A (*MeCaM1* and *MeCaM4*) were clustered together with CaMs from *Arabidopsis* and rice. All of these genes are broadly expressed in various tissues of cassava, *Arabidopsis*, and rice (Figure 4B) [51,52]. Besides, some CMLs and CBLs with close phylogenetic relationship among cassava, *Arabidopsis*, and rice also showed similar expression profiles in different tissues [52,53]. This indicated a possible link between sequence conservation and gene expression for the CaMs, CMLs, and CBLs.

Accumulated evidences have documented the positive role of Ca^2+^ sensors in drought stress response [11,54,55,56,57]. In the present study, we found that some cassava *MeCaMs-MeCMLs-MeCBLs* were upregulated by drought stress in roots and leaves of different genotypes, suggesting the possible role of Ca^2+^ sensor genes in cassava response to drought stress (Figure 4C). Additionally, the number of *MeCaMs-MeCMLs-MeCBLs* induced by drought is greater in Arg7 than in SC124 and W14, suggesting the comprehensive activation of *MeCaMs-MeCMLs-MeCBLs* in the Arg7 variety (Figure 4C). Arg7 was confirmed to be a drought-sensitive variety, which showed lower tolerance to drought than W14 and SC124 [24]. It is concluded that Arg7 variety has strong response to drought stress, thus leading to the comprehensive transcriptional induction of *MeCaMs-MeCMLs-MeCBLs*. Interestingly, the number of *MeCaMs-MeCMLs-MeCBLs* induced by drought was greater in roots than in leaves in W14, whereas it was reduced in roots compared with leaves in Arg7 and SC124 (Figure 4C). Cassava maintains its robust drought tolerance through absorbing water stored in the deeper soil layers by the deep root systems [58]. Thus, it is speculated that significant induction of *MeCaMs-MeCMLs-MeCBLs* in roots of W14 may be involved in water uptake, thus benefit for maintaining its strong tolerance to drought stress.

During cassava harvest and storage, SR deterioration occurrence is difficult to prevent due to storage conditions, handling operations, and mechanical injury [29]. Physiological and genetic evidences revealed that oxidation caused by reactive oxygen species (ROS) lead to the SR deterioration symptoms, and reduction of ROS accumulation could attenuate SR deterioration [27,29,59,60]. Calcium, an important signaling molecular, plays a protective role in plants response to abiotic stress [61]. One of the important events underlying this protection is activating antioxidative system and repressing ROS accumulation [62,63,64,65]. These studies suggested that calcium signaling may play a role in regulating SR deterioration by influencing cellular ROS level. In the present study, we found that numerous calcium sensor genes were significantly induced in cross-sessions of SR at 6 h, 12 h, and 48 h compared with 0 h after harvest (Figure 4D). Further interaction network and co-expression analyses identified some gene pairs uniformly upregulated at 48 h/0 h in cross-sessions of SR after harvest (Figure 5). These results indicated the possible involvement of calcium sensors during SR deterioration of cassava. Based on the transcriptomic data, there were some Ca^2+^ sensor genes that showed induction after drought treatment and during SR deterioration, including *MeCML-4*, *-10*, *-16*, *-17*, *-20*, *-22*, *-25*, *-26,* and *MeCBL2*. These genes can serve as candidates for genetic improvement of cassava resistance to drought and SR deterioration.

## 5. Conclusions

This study identified 12 *MeCaMs*, 56 *MeCMLs*, and 9 *MeCBLs* from the cassava genome, and investigated their classification, protein motif, and gene structure. Transcriptome analysis showed the possible role of *MeCaMs-MeCMLs-MeCBLs* against drought stress and in cross-sessions of SR deterioration after harvest. Interaction network and co-expression analyses revealed the involvement of a CBL-mediated network in SR deterioration after harvest. Together, these results will advance the understanding of calcium signaling-mediated drought stress response and SR deterioration regulation in cassava.

## Supplementary Materials

The following are available online at http://www.mdpi.com/2073-4425/9/4/221/s1. Figure S1: Interaction network and co-expression analyses of CAMs (A) and CMLs (B) at 48 h/0 h after harvest, Table S1: The accession number of transcriptomic data in NCBI, Table S2: Characteristics of CaMs, CMLs, and CBLs in cassava, Table S3: The accession numbers of CaMs, CMLs, and CBLs in *Arabidopsis* and rice, Table S4: Expression of cassava CaMs, CMLs, and CBLs in different tissues of Arg7 and W14, Table S5: Expression of cassava CaMs, CMLs, and CBLs in response to drought stress in Arg7, SC124 and W14, Table S6: Expression of cassava CaMs, CMLs, and CBLs during SR deterioration, Table S7: The protein interaction relationship in the CaM-mediated interaction network in *Arabidopsis*, Table S8: The protein interaction relationship in the CML-mediated interaction network in *Arabidopsis*, Table S9: The protein interaction relationship in the CBL-mediated interaction network in *Arabidopsis*, Table S10: Expression data of the genes involved in CaM/CBL/CML-mediate interaction networks at CK48/CK0 after harvest, Table S11: The co-expression patterns of gene pairs involved in CAM/CBL/CML-mediate interaction networks at 48 h/0 h after harvest, Table S12: The accession number of the genes in the CBL-mediated interaction network.
