# Supplementary figures and images for: Genome-Wide Analyses of Calcium Sensors Reveal Their Involvement in Drought Stress Response and Storage Roots Deterioration after Harvest in Cassava
Source: Genes (Basel). 2018 Apr 19;9(4):221. doi: 10.3390/genes9040221 (PMC5924563; doi:10.3390/genes9040221)

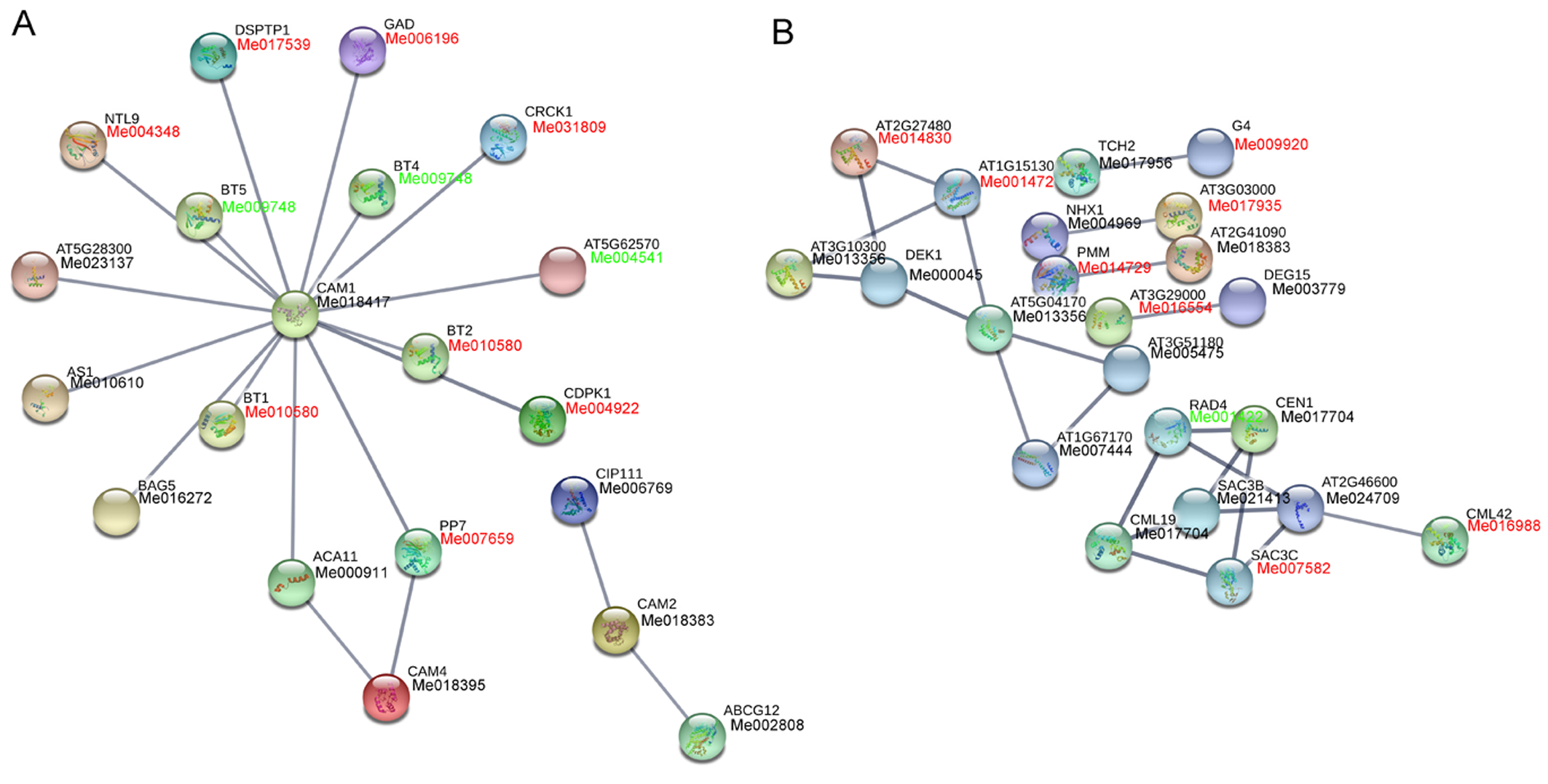

Supplement: Supplementary file 1 [file genes-09-00221-s001.zip › Figure S1.tif]
